# Hydrogen Bonds in Lead Halide Perovskites: Insights from *Ab Initio* Molecular Dynamics

**DOI:** 10.1021/acs.jpcc.3c02376

**Published:** 2023-08-08

**Authors:** Alejandro Garrote-Márquez, Lucas Lodeiro, Rahul Suresh, Norge Cruz Hernández, Ricardo Grau-Crespo, Eduardo Menéndez-Proupin

**Affiliations:** † Departamento de Física Aplicada I, Escuela Politécnica Superior, Universidad de Sevilla, Seville E-41011, Spain; ‡ Departamento de Química, Facultad de Ciencias, Universidad de Chile, Las Palmeras 3425, Santiago, Ñuñoa 7800003, Chile; § International Research Center of Spectroscopy and Quantum Chemistry - IRC SQC, Siberian Federal University, 79 Svobodny pr., 660041 Krasnoyarsk, Russia; ∥ Department of Chemistry, Whiteknights, University of Reading, Reading RG6 6DX, UK

## Abstract

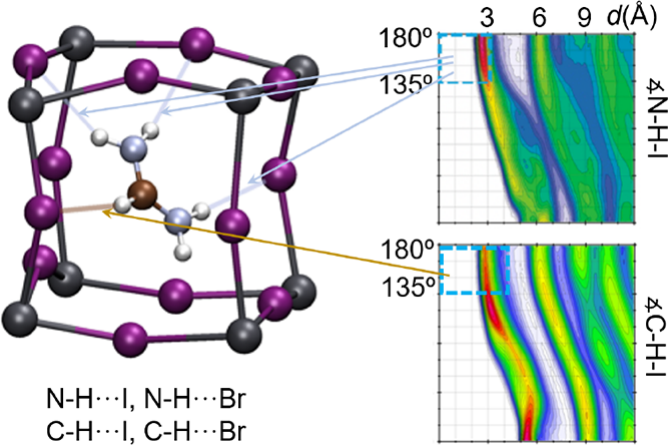

Hydrogen bonds (HBs) play an important role in the rotational dynamics of organic cations in hybrid organic/inorganic halide perovskites, thus affecting the structural and electronic properties of the perovskites. However, the properties and even the existence of HBs in these perovskites are not well established. In this study, we investigate HBs in perovskites MAPbBr_3_ (MA^+^ = CH_3_NH_3_^+^), FAPbI_3_ (FA^+^ = CH(NH_2_)_2_^+^), and their solid solution with composition (FAPbI_3_)_7/8_(MAPbBr_3_)_1/8_, using *ab initio* molecular dynamics and electronic structure calculations. We consider HBs donated by X-H fragments (X = N and C) of the organic cations and accepted by the halides (Y = Br and I) and characterize their properties based on pair distribution functions and on a combined distribution function of the hydrogen–acceptor distance with the donor–hydrogen–acceptor angle. By analyzing these functions, we establish geometrical criteria for HB existence based on the hydrogen–acceptor (H–Y) distance and donor–hydrogen–acceptor angle (X–H–Y). The distance condition is defined as *d*(H – Y) < 3 *Å* for N–H-donated HBs and *d*(H – Y) < 4 *Å* for C–H-donated HBs. The angular condition is 135^°^ < 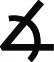 (X – H – Y) < 180^°^ for both types of HBs. A HB is considered to be formed when both angular and distance conditions are simultaneously satisfied. At the simulated temperature (350 K), the HBs dynamically break and form. We compute the time correlation functions of HB existence and HB lifetimes, which range between 0.1 and 0.3 ps at that temperature. The analysis of HB lifetimes indicates that N–H–Br bonds are relatively stronger than N–H–I bonds, while C–H–Y bonds are weaker, with a minimal influence from the halide and cation. To evaluate the impact of HBs on the vibrational spectra, we present the power spectrum in the region of N–H and C–H stretching modes, comparing them with the normal mode frequencies of isolated cations. We show that the peaks associated with N–H stretching modes in perovskites are redshifted and asymmetrically deformed, while the C–H peaks do not exhibit these effects.

## 1 Introduction

Hybrid organic–inorganic halide perovskites (HOIHPs) constitute a versatile family of materials that have attracted much research attention during the last decade. Since first proposed in 2009 by Kojima et al.,^1^ perovskite solar cells have emerged as a strongly promising candidate for efficient and cheap solar cells and have attained an extraordinary 26.0% record photoconversion efficiency (PCE) as single junction solar cells and 33.7% in tandem perovskite/silicon solar cells.^2^ Other promising applications of HOIHPs include X-ray detectors,^3^ LED devices,^4^ and water splitting photocatalysts.^5^ HOIHPs are flexible in terms of morphology and can be synthetized as bulk crystals, bidimensional laminar crystals, or quantum dots.

**Figure 1:**
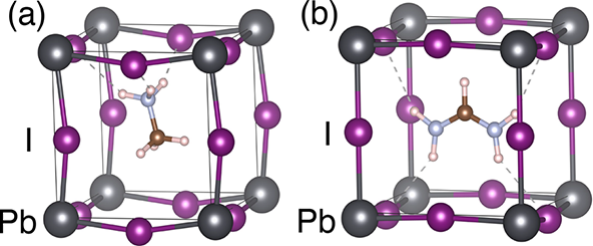
Representation of MAPbI_3_ (a) and FAPbI_3_ (b) perovskite structures. Hydrogen bonds are indicated by thin dashed lines. Atoms are represented by balls in colors: gray (Pb), violet (I), brown (C), blue (N), and white (H). FAPbI_3_ structure from ref (7). Images created with VESTA.^8^

The crystal structure of 3D halide perovskites, with the general formula ABY_3_, is well illustrated by methylammonium lead iodide (CH_3_NH_3_PbI_3_), represented in Figure 1a. The A-site of perovskite is occupied by the organic cation methylammonium (CH_3_NH_3_^+^), the B-site is occupied by the lead cation (Pb^2+^, gray balls), and the Y-sites are occupied by the iodide anion (I^–^, violet balls). Methylammonium is often abbreviated as MA or MA^+^, leading to the short names MAPbI_3_ or MAPI for the compound CH_3_NH_3_PbI_3_. Other members of the family of 3D HOIHPs can be generated, replacing the MA by another organic cations like formamidinium (FA), CH(NH_2_)_2_ (Figure 1b shows the structure of FAPbI_3_), or by a large inorganic cation like cesium; the iodide anion can be also replaced by bromide or chloride, and the lead cation can be replaced by tin or germanium. The full family includes solid solutions of the pure compounds, where each crystal site can be occupied randomly by one or several of the above-described components. These mixed compounds show a remarkable improvement in their stability (the HOIHP’s Achilles heel) and are related with the evolution of record PCE.^6^

The perovskite structure is generally stable when the ionic radii *r*_A_, *r*_B_, *r*_Y_ satisfy an empirical rule 0.8 < *t* < 1.0, according to the Goldschmidt tolerance factor:^9^
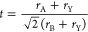


The nature of the A-site cation has a dramatic influence on the phase stability and on crystal symmetry.^10^ Substitution of the A-site cation by organic cations larger than FA, with *t* > 1.0, limits crystal growth and allows the formation of laminar structures (2D perovskites),^11^ which are periodic in two directions and are a few unit cells wide in the third direction. Another example is CsPbI_3_, which is stable in the perovskite structure at temperatures just over 600 K, but when cooled to room temperature, it makes a fast transition to a yellow non-perovskite phase. A similar behavior is exhibited by FAPbI_3_; however, their solid solution, Cs_*x*_FA_1–*x*_PbI_3_, is stable at room temperature.^6^ Perovskite phases show a diversity of space groups and phase transitions at different temperatures, depending on the organic cation,^10,12^ as well as on the B-site cation^10^ and on the halide.^13^

As Figure 1 illustrates, the inorganic PbY_3_ backbone presents large distortions from the ideal perovskite structure. The cells shown in this figure represent formula units of the HOIHP but do not possess the crystal symmetries inferred from diffraction techniques. Time- and space-dependent realizations of these basic cells conform the average structures that make the apparent crystal periodicity in diffraction experiments. Higher-temperature perovskite phases display an apparent cubic symmetry, where the crystallographic unit cell coincides with those of Figure 1, but that represents only the average positions of the atoms, with large thermal ellipsoids. Moreover, the organic cations rotate and make orientational jumps across all the possible equivalent orientations. For the lower-temperature perovskite phases, the primitive cells are supercells of those represented in Figure 1. For example, MAPbI_3_ undergoes a cubic to tetragonal transition at ∼330 K, and the primitive cell has two formula units, and a tetragonal to orthorhombic transition at ∼160 K with a primitive cell of four formula units. In the latter case, the cations do not rotate, and all atoms keep at definite positions.

Hydrogen bonds (HBs) are possible in HOIHPs between the halides Y^–^ and the protons bound to nitrogen or carbon atoms, as indicated by dashed lines in Figure 1. HBs may influence the rotational dynamics of the organic cations and therefore play a role in the stabilization of the HOIHP, the deformations of the inorganic backbone, and indirectly on the electronic structure.^9,14−23^ The understanding and characterization of HBs in perovskites have therefore attracted research attention.

**Figure 2:**
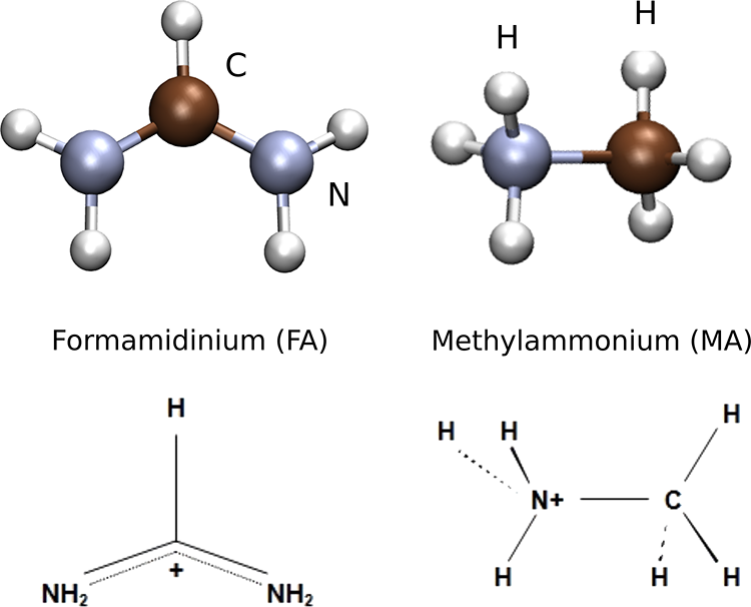
Formamidinium (FA) and methylammonium (MA) cation geometry (top) and Lewis structure (bottom). The location of positive charge is indicated, as determined by the extra proton and the electronic cloud distribution.

Figure 2 illustrates the structure of FA and MA. Methylammonium is a result of the protonation of methylamine neutral molecule (H_2_N–CH_3_), where the proton is attracted by the lone pair of the H_2_N group. The protonated ammonium group (H_3_N) has three equivalent N–H bonds; therefore, the positive charge^24^ is considered a property of the full group rather than localized at any of the hydrogen atoms. Hence, the hydrogens of the ammonium extreme are selectively attracted to the halide anions in the perovskites. FA is a result of formamidine (H_2_N–CH=NH) protonation. In formamidine, the central carbon atom is involved in a single bond with a nitrogen atom and a double bond with the other nitrogen atom, leaving a lone pair at the latter. However, upon protonation at the NH group, the structure becomes resonant between the Lewis structures H_2_N–CH=NH_2_ and H_2_N=CH–NH_2_. FA has a planar structure, as shown in MD simulations, which can be attributed to the double-bond character between the carbon and both nitrogen atoms. The delocalization of the double bond toward the incoming proton can be understood as a positive charge density distributed along the N–C–N backbone, as shown in Figure 2. Hence, the attraction exerted by halide anions upon each hydrogen should be smaller than in the MA case due to this distribution of the positive charge. We consider first the HBs donated by the NH_2_ groups, but later we will also consider the possibility of donation by the CH group.

Svane et al.^25^ devised a method to compute the HB energies in hybrid perovskites, filtering out the electrostatic interaction of the charged organic cation with the inorganic lattice. For halide perovskites, they obtained energies in the range of 0.02 to 0.27 eV per organic cation, which is one order of magnitude smaller than in formate perovskites. In the high-temperature phases of HOIHPs, the HBs are not permanent but form and break dynamically, allowing cation rotation and determining the symmetry of the crystal. The existence of HBs in lead-based HOIHPs at room temperature has been recently questioned by Ibaceta-Jaña et al.^26^ based on a combined Raman and density functional theory (DFT) study.

The question of the existence of HBs at given conditions requires a careful definition of these bonds. According to IUPAC recommendations,^27,28^ HBs should display some charge transfer, show directional preference, and show a bond path connecting the hydrogen with the HB acceptor. Early conceptions of HBs considered only donors like F–H, O–H, and N–H, the last one present in HOIHPs. However, the list of donors has expanded to include any molecule or molecular fragment X–H, where X is any element with electronegativity larger than H, i.e., F, N, O, C, P, S, Cl, Se, Br, and I.^28^ The acceptor Y can be any of these elements. Therefore, the HB donors in HOIHPs can be the N–H and C–H groups of the organic cations, while the acceptors can be the halides Cl, Br, and I. Directionality is an important criterion; the angle X–H–Y should be close to 180°. Vibrational spectroscopy provides a signature of HBs. The X–H stretching modes typically decrease its frequency (although a blue shift is also possible), accompanied by an increase of bandwidth and change of intensity in the IR spectra. The X–H peak can even disappear from the spectrum, although this is understood as a large redshift and mixing with other modes.^28^ The HBs also modify the topology of electron density, obtained either from experiments or from electronic structure calculations. In particular, the electron density must present a (3, −1) critical point along the H–Y direction, meaning that the gradient of the density is maximum (minimum) along two (one) orthogonal directions. Methods based on the electron density topology are available to identify HBs using quantum chemistry calculations.^21,29,30^ Finally, HBs need to be thermally stable to have practical significance, but the absence of effects does not deny its very existence as temporary states.

In this work, we present a systematic theoretical study of the HB features for three HOIHPs, MAPbBr_3_, FAPbI_3_, and the solid solution (FAPbI_3_)_7/8_(MAPbBr_3_)_1/8_, using *ab initio* molecular dynamics (AIMD). For these compounds, we will use the short names MAPBr, FAPI, and MAFA, respectively. We will present statistical functions related to the HBs, such as radial distribution functions, combined (radial and angle) distribution functions, autocorrelation functions, and lifetimes, to address some of the open questions about HBs in halide perovskites. This work builds upon the model of MAFA solid solution presented in ref (31) and the AIMD simulations developed there for MAFA, MAPBr, and FAPI. In the present study of HBs, we have imported methods and codes from the field of ionic liquids, explaining the methodology and the numerical parameters (cutoff distances and angles) that are appropriate to define the HBs in HOIHPs.

One important precedent is ref (20), where the HBs in pure MAPBr were studied using classical molecular dynamics (MD), i.e., with parameterized force fields. The latter method, although generally less accurate than AIMD, allowed the authors to include a large number of atoms in the simulation and perform an extensive statistical study, focusing on properties different from those considered in our study, such as HB energetics, spatial correlations of cation orientation, and its influence on the electronic structure. Notably, the directionality of the HB^27,28^ was not considered in ref (20), which led them to overestimate the HB population and probably its importance.

In contrast, Ibaceta-Jaña et al.^26^ claimed that no HBs exist at room temperature in HOIHPs, or at least they cause no observable effects in the infrared and Raman spectra. To shed some light in this issue, we also present the calculations of the power spectra that show that HBs may have observable effects in the region of highest frequencies, not covered by the experiments of ref (26). We also present the calculations of the reduced density gradient that reveal HB effects on the electronic structure of certain temporary states.

## 2 Methods

The (FAPbI_3_)_7/8_(MAPbBr_3_)_1/8_ solid solution is represented by a special quasi-random structure, with an ion distribution that mimics the random distribution in terms of short-range pair correlation functions, as described in previous work.^31^ The coordinate files of selected configurations are available in ref (31), while the full MD ensemble is deposited in a free repository.^32^ Our AIMD simulations at constant volume and temperature (NVT ensemble), with *T* = 350 K, were carried out using the CP2K package.^33^ The ionic forces were calculated using DFT in the generalized gradient approximation as implemented in the Perdew–Burke–Ernzerhof (PBE) functional,^34^ with the Grimme correction scheme DFT-D3^35^ to account for the dispersion interactions. The hybrid Gaussian and plane wave method^36^ implemented in the QUICKSTEP module of the CP2K package was used. The Kohn–Sham orbitals of valence electrons are expanded in a Gaussian basis set (DZVP-MOLOPT for Pb, I, Br, C, N, and H).^37^ Core electrons were treated using dual-space GTH pseudopotentials.^38−40^ Further details of the AIMD calculations can be found in ref (31).

The TRAVIS code^41^ was used to characterize the HBs via pair distribution functions (PDFs), combined distribution functions (CDFs), and time correlation functions.^41^ The PDF, also called the radial distribution function, gives the probability that two atoms of different species are separated a given distance, relative to the probability in a non-interacting gas; the distance of separation is the argument of the function. The PDF reveals the short-range order of a liquid or a disordered material; bond lengths are identified from the peaks at the shortest distances, and coordination spheres are identified from the minima of the PDF. TRAVIS also allows us to obtain other distributions like angle distribution functions and dihedral distribution functions. The CDFs are combinations of the former distributions, providing the probability of finding certain combinations of geometrical parameters, such as the pair distance and the angle between three atoms. In particular, the CDF of the halide–hydrogen distance and the halide–hydrogen–nitrogen angle allows to reveal the HBs in HOIHPs. The HB dynamics can be characterized by the time correlation functions of the pair forming the HB:
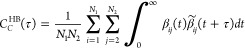
1where β_*ij*_(*t*) = 1 if there is a HB between atoms *i* and *j* (one halide and one hydrogen) or zero otherwise. The condition of existence of a HB is defined in terms of a distance and an angle, as will be discussed below. The function β̃_*ij*_(*t* + τ) = 1 if the HB exists for all the time between *t* and *t* + τ, and is zero if the HB breaks at any instant. This is called the continuous autocorrelation function in TRAVIS. The function *C*_*C*_^HB^(τ) decays to zero, and the lifetime of the HB can be obtained as
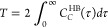
2

TRAVIS also allows us to compute intermittent autocorrelation functions, where β̃_*ij*_(*t* + τ) is replaced by β_*ij*_(*t* + τ), which equals unity if the HB exists at *t* + τ, regardless of whether it breaks or not at intermediate times. The intermittent autocorrelation function is useful for HBs in liquids but not in HOIHPs because the organic cations are confined in a cavity and any broken HB will form again and again, not allowing the autocorrelation function to decay to zero. However, this kind of function has been used in ref (23), allowing to extract approximate lifetimes from the initial fast decay.

## 3 Results

### 3.1 HB Donated by Ammonium and Amidine Groups

**Figure 3:**
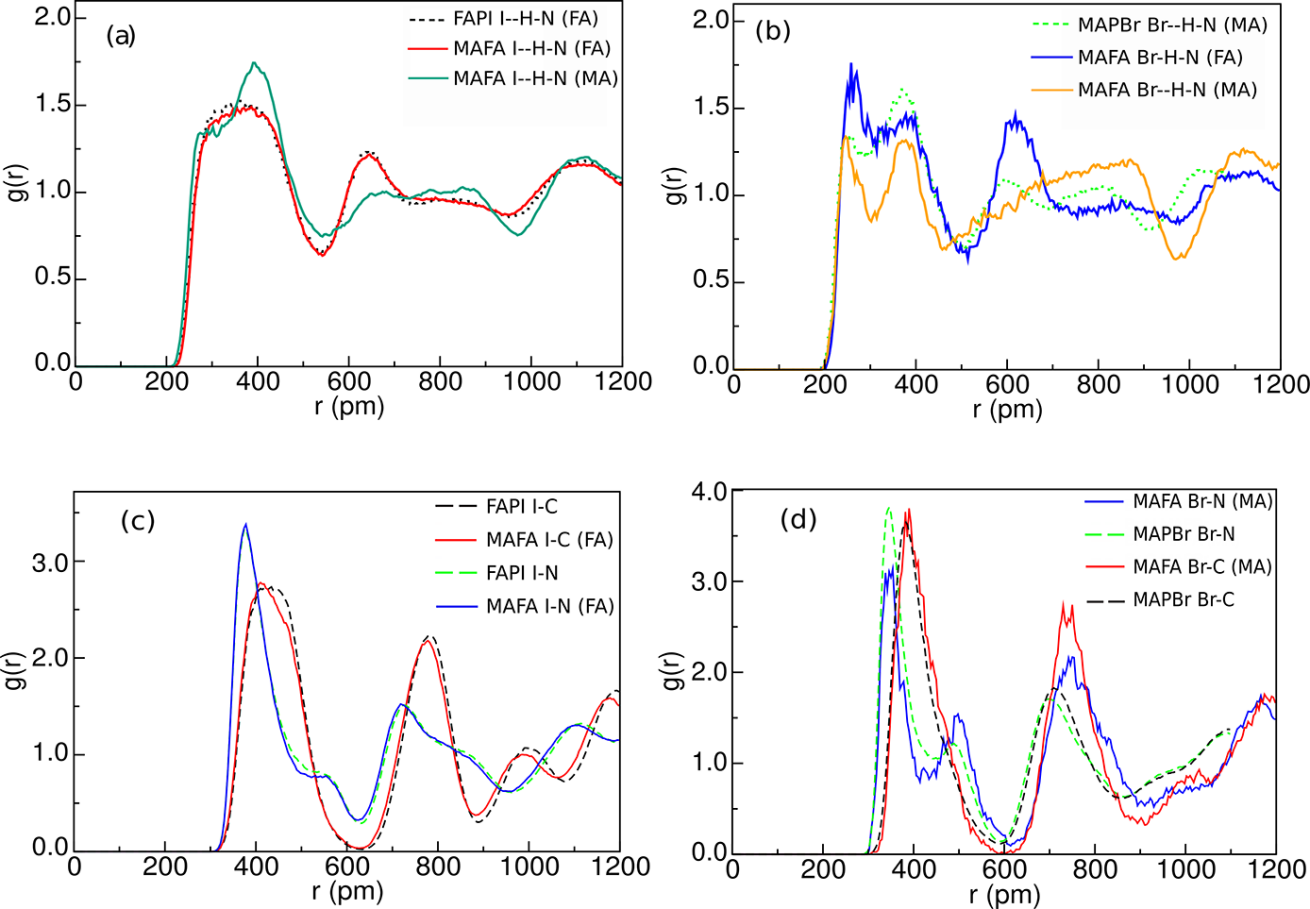
PDFs for (a) iodide–hydrogen, considering hydrogen covalently bound to nitrogen in MAPBr, FAPI, and MAFA (short name for (FAPbI_3_)_7/8_(MAPbBr_3_)_1/8_); (b) bromide–hydrogen; (c) iodide–carbon/nitrogen; and (d) bromide–carbon/nitrogen.

Figure 3a,b shows the PDFs Y–H–N(A) as functions of the halide–hydrogen distance Y–H in FAPI, MAPBr, and MAFA. Here, Y = Br or I, and A = MA or FA, the latter indicating the cation the nitrogen belongs to. The dotted curves represent the distribution functions present on the pure compounds, i.e., Br–H–N(MA) in MAPBr and I–H–N(FA) in FAPI. The corresponding functions for MAFA are shown in solid lines, as well as for the pairs present only in MAFA, i.e., I–H–N(MA) and Br–H–N(FA). The HB is signaled by the sharp peaks between 2 and 3 Å, except for I–H–N(FA) in FAPI (black dotted line) and MAFA (red line). A subset of these functions, those present in pure compounds, were presented in ref (31), showing that Y–H–N distances are shorter than Y–H–C distances. The latter case (hydrogen covalently bound to carbon) will be analyzed later. Figure 3c,d shows that halide–nitrogen distances are smaller than halide–carbon distances. The finding that the Y–N and Y–H–N distances are shorter than the Y–C and Y–H–C distances, respectively, has been reported for MAPI elsewhere.^15,17^ This behavior can be expected from the positive charge of the NH_3_ group in MA. This argument does not apply to FA, as the positive charge is delocalized along the N–C–N backbone. Instead, the elongated shape of FA favors the N atoms at the edges getting closer to the halides. Other remarkable property is that the Y–C PDFs have minima with null or almost null value at ∼6 Å, coincident with the lattice parameters (6.3620, 6.3115, and 5.9328 Å for FAPI, MAFA, and MAPBr, respectively). The corresponding minima of Y–N PDFs are not that sharp but are still well defined. In contrast, the first minima Y–H–X PDFs are still higher than 0.5. This is a consequence of H atoms being distributed in large rigid cations. However, as we will see next, sharp minima are obtained when directionality criteria are incorporated.

**Figure 4:**
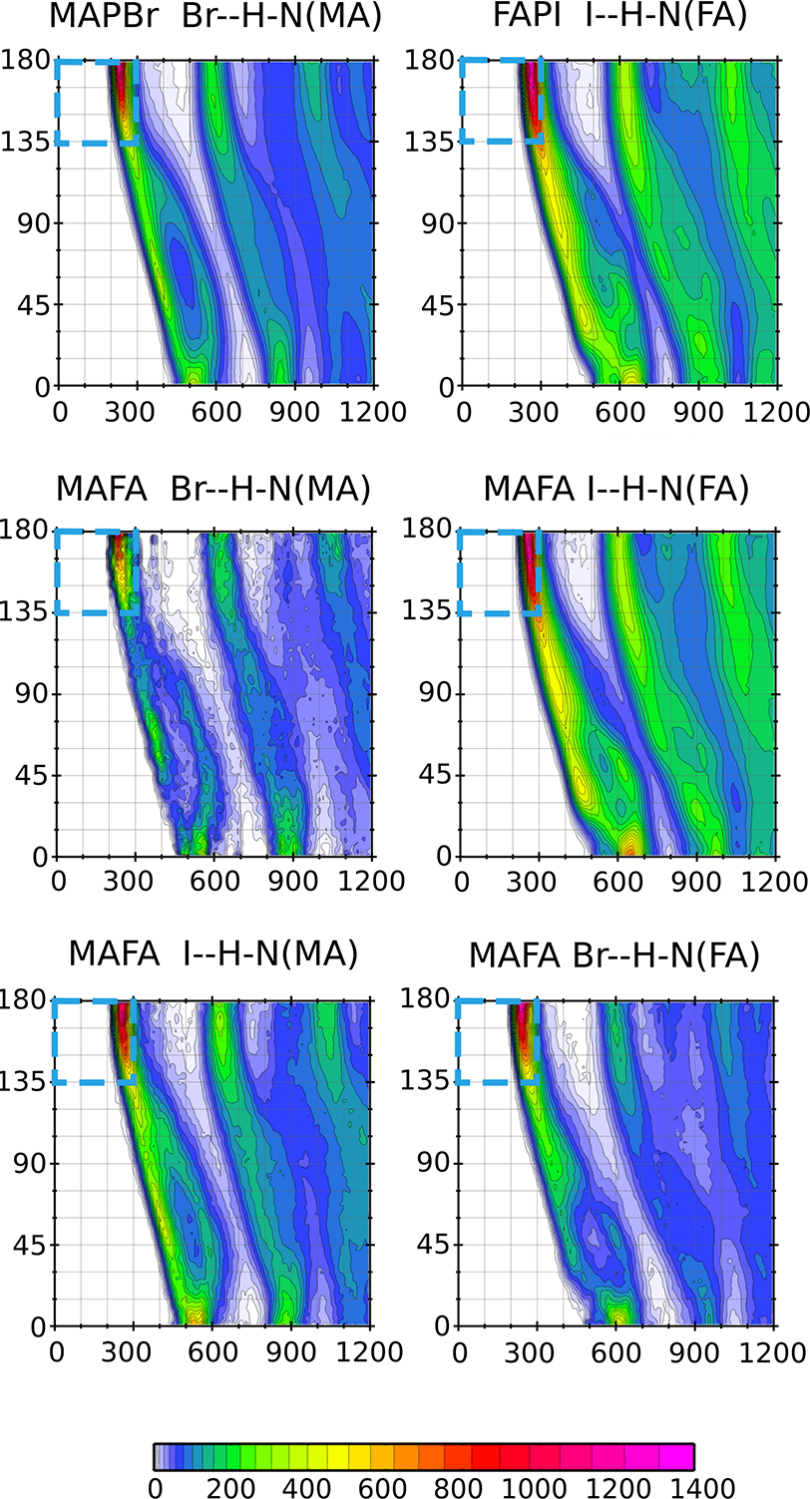
CDFs of the Y–H distance (Y = Br and I) in pm (horizontal axes) with the 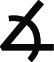
(Y – H – N) angle in degrees (vertical axes) for the pure compounds and the solid solution.

HBs are better resolved by means of CDFs, which are functions of the Y–H distance and the Y–H–N angle. Figure 4 shows the CDF for the same combinations as in Figure 3a. The maximal values take place in the region of the red spots, which can be approximately defined by the simultaneous conditions *d* < 3 *Å* and 135^°^ < 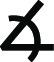 (I – H – N) < 180°. Even for I–H–N(FA) in FAPI and MAFA, which do not show a peak in the PDF, the CDF shows a clear maximum at distances between 2 and 3 Å, followed by minimum between 4 and 5 Å, provided that the angle is larger than 135°. Therefore, we take this couple of geometrical conditions as the definition for the existence of a HB.

**Figure 5:**
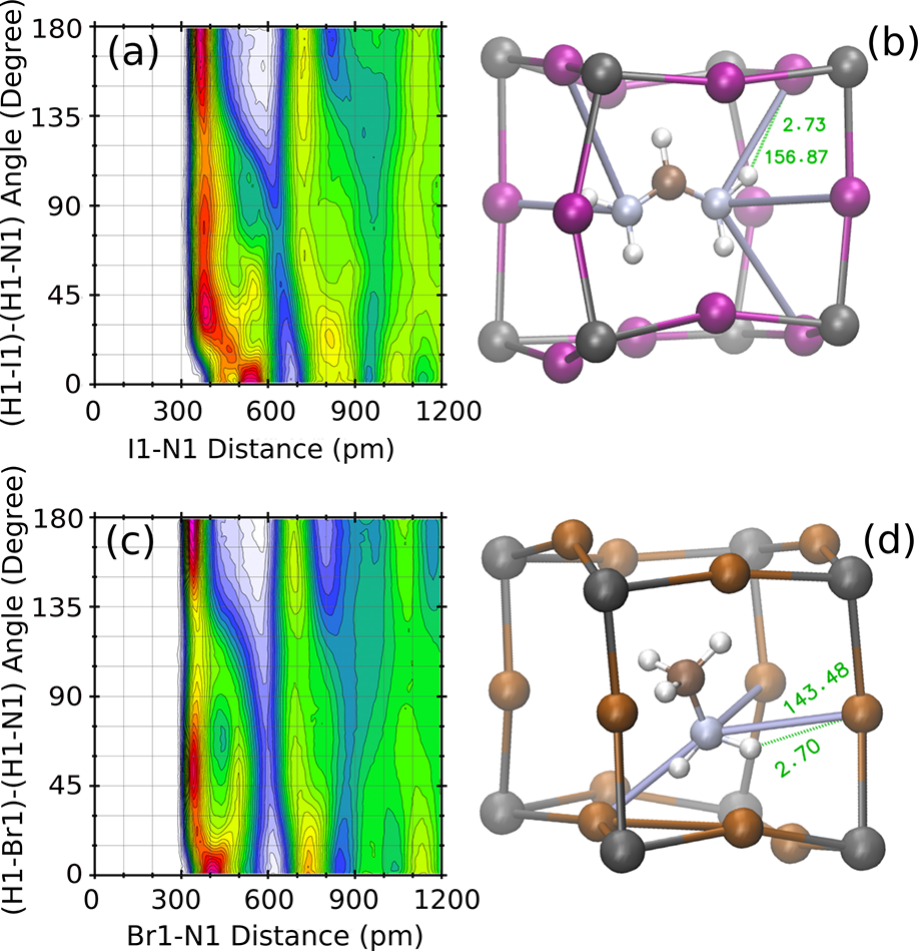
Left: CDF of the Y–N distance and Y–H–N angle (Y = I and Br) in FAPI (a) and MAPBr (c). Right: Snapshot of the MD illustrating FAPI (b) and MAPBr (d) with one “real” HB and several fake HBs defined by just Y–N distances less than 4 Å. The meaning of labels Br1, H1, N1, and I1 is explained in the Supporting Information.

The animation videos V1 to V6 in the Supporting Information show that, with these conditions, HBs form and break dynamically during the simulation time. These animations were generated with the Visual Molecular Dynamics (VMD) graphics program,^42^ where HBs are defined somewhat differently. HBs in VMD are defined by the Y–N distance instead of the Y–H distance and the same condition for the angle 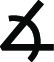
(Y – H – N). For these videos, the Y–N distance cutoff was set as 4 Å, i.e., 1 Å larger than the Y–H distance cutoff that we have explained above. It is tempting to derive this condition from the CDF of the Y–N distance and Y–N–H angle. This CDF, shown for FAPI and MAPBr in Figure 5a,c, shows the maxima for distances between 3 and 4 Å, but it is almost independent of the angular condition. The snapshots in Figure 5b,d show the Y–N distances smaller than 4 Å (sticks in gray color). Using just the distance condition would imply HBs with up to three halide atoms simultaneously. However, HBs defined by the combination of distance and angle conditions allow HB bonds with single halides, as shown by the lines in green color. For the HB shown in Figure 5b, the distance I–H and the angle I–H–N are 2.73 Å and 156.87°, respectively. Similarly, for the HB shown in Figure 5d, the distance Br–H and the angle Br–H–N are 2.70 Å and 143.48°, respectively.

The animation video V7 in the Supporting Information complements this view, indicating that the first peaks of the Y–N PDF seen in Figure 3b,c are not explained by HB formation. Proximity of nitrogen and iodine in FAPI has instead steric origin due to the cation size and the location of N atoms at opposite sites. In the case of MA, the peak of the Br–N PDF also has only a partial contribution from HBs. The smaller cation size and the presence of only one N atom suggest that this peak is caused by the electrostatic attraction between the halide and the NH_3_^+^ group and HB. However, the region in the CDF of Figure 5a,c, corresponding to 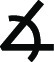
(Y – H – N) > 135^°^, shows a depletion for the Y–N distance between 5 and 6 Å. This is the signature of HB in this CDF, explaining why the HB can be visualized in VMD by defining the HB through the angular condition together with the Y–N distance, although VMD displays a dotted line along the H–Y path. The same behavior can be appreciated for the solid solution MAFA in Figure S1 of the Supporting Information.

**Figure 6:**
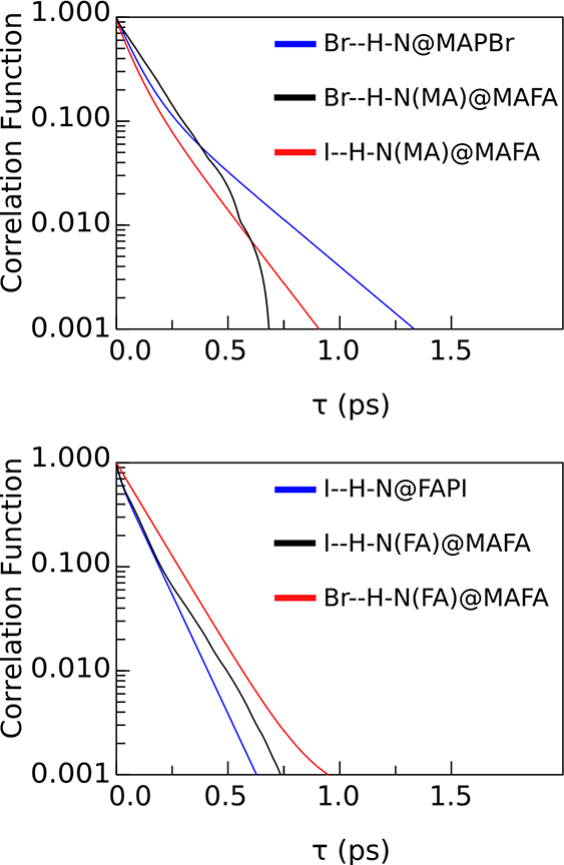
Autocorrelation functions of all the Y–H–N bonds studied.

After the geometrical definition of HBs has been established, we now probe their dynamics by means of autocorrelation functions, as defined in eq 1, and the lifetimes, as defined from eq 2. Based on the autocorrelation functions (Figure 6), we present the HB lifetimes in Table 1. The lifetimes are dependent on the limits set to the distance and angle, but given a common definition, we can observe several trends. One can appreciate that the Br–H–N(MA) and Br–H–N(FA) have the longest duration. Also, it is apparent that the Br–H–N(MA) bond increases its lifetime in the MAFA solid solution (0.27 ps) with respect to MAPBr. However, there are very few Br and few MA in the MAFA model, which leads to poor statistics. The value 0.27 ps is the average of the lifetimes of three symmetrically equivalent hydrogen atoms: two of them have a lifetime of 0.23 ps and the third one has a lifetime of 0.34 ps. The set of all lifetimes, separated by different hydrogens and nitrogens, is shown in Table S2 of the Supporting Information. The HB with iodine has shorter lifetimes than the HB with bromine. Table 1 also includes the lifetimes of HBs with hydrogens attached to carbon atoms, which will be discussed in the next section.

**Table 1:** Lifetimes (in ps) of the Different Types of HBs Derived from the HB Continuous Time Correlation Functions

	FAPI	MAPBr	MAFA
I–H–N(MA)			0.18
Br–H–N(MA)		0.23	0.27
I–H–N(FA)	0.15		0.15
Br–H–N(FA)			0.23
I–H–C(MA)			0.14
Br–H–C(MA)		0.16	0.15
I–H–C(FA)	0.15		0.16
Br–H–C(FA)			0.18

### 3.2 HB through Carbon Atoms

We also explore the possibility of the HB donated by the C–H groups of MA and FA. Deprotonated FA can be formamidine (H_2_N–CH=NH) or diaminocarbene (H_2_N–C–NH_2_). The latter is highly unstable, but it has been found in mass spectroscopy experiments.^43^ Regarding HOIHPs, several authors^21,44^ have studied the role of Y–H–C bonds, among several non-covalent interactions, and they have pointed the importance of HBs in determining the extent of the PbI_6_ octahedra tilting in the low-temperature phases. Hence, we proceed to study this possible HB with the same approach used for Y–H–N bonds.

**Figure 7:**
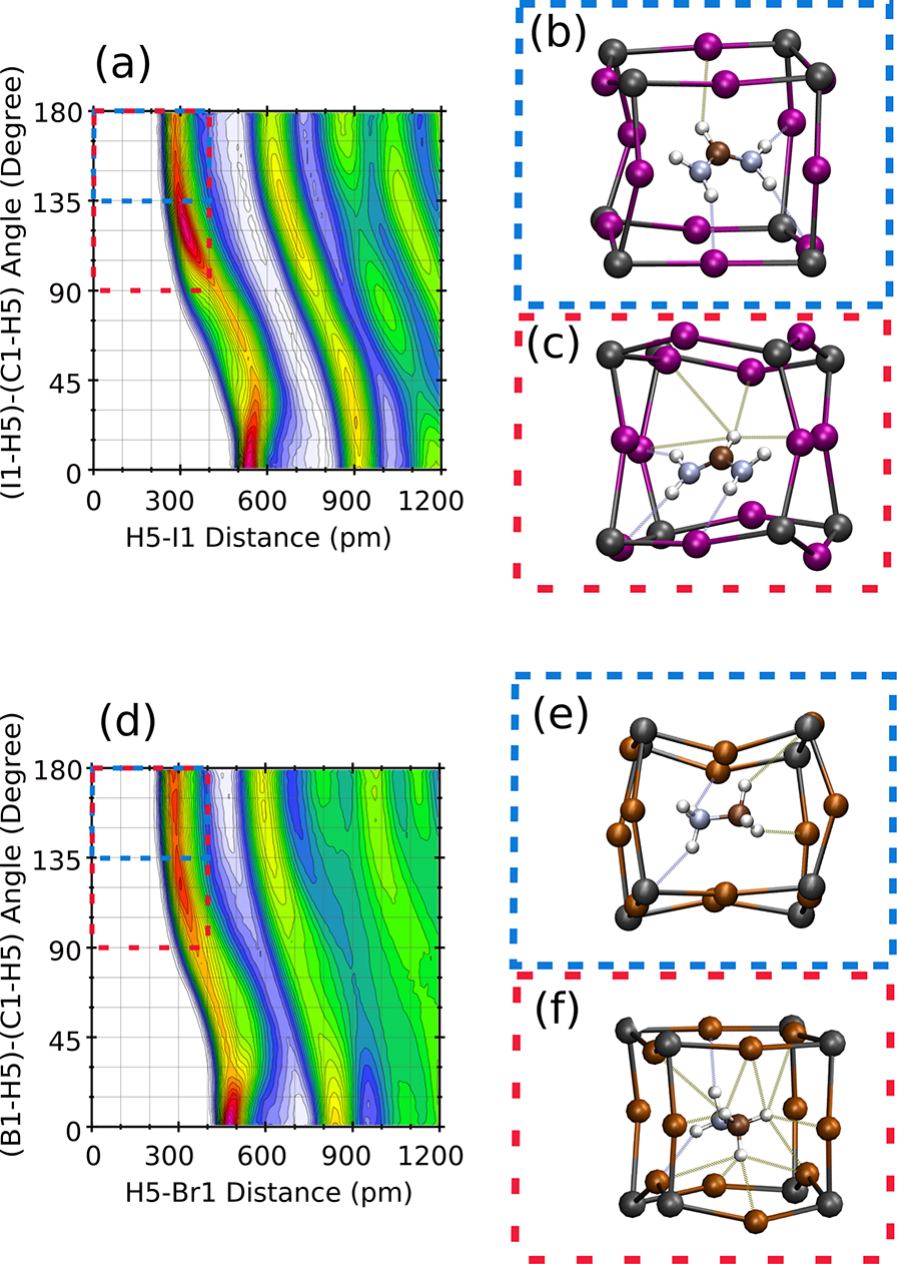
Illustration of the HB with carbon and nitrogen atoms for FAPI (a–c) and MAPBr (d–f). Images b, c, e, and f are snapshots of the MD simulations, showing the HB by ghostly lines. The CDFs (only for Y–H–C angles) for FAPI (a) and MAPBr (d) indicate the range of distances and angles by blue rectangles. The plots at the left (right) side indicate a wide (narrow) range of X–H–C and X–H–N angles. The meaning of labels Br1, H5, C1, and I1 is explained in the Supporting Information.

The CDF of the I–H distance with the I–H–C angle, shown for FAPI in Figure 7a, suggests that the HB I–H–C(FA) could be defined from the conditions *d*(I – H) < 4 *Å* and 90^°^ < 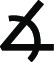 (I – H – C) < 180^°^ . However, inspection of the MD animation (see video V8) shows that this geometric condition leads frequently to the HB of one H with several iodine atoms simultaneously, as shown in Figure 7c. Considering a more restrictive condition for the angle (Figure 7a) like that used to define I–H–N bonds, i.e., 135^°^ < 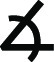 (I – H – C) < 180^°^, a single bond is appreciated, as in Figure 7b (also compare supporting video V8 with V1). Let us annotate that the configuration of Figure 7c has no HB with the restricted conditions of Figure 7b. Moreover, a distance cutoff of 4 Å suggested by the CDF is 1 Å larger than the cutoff for the I–H–N(FA) distance (from the CDF shown in Figure 4). Bond distances larger for I–H–C(FA) than for I–H–N(FA), as observed from the location of the red spots in the CDFs of Figures 4 and 7, respectively, are usually related with a softer bond. Figure 7d–f illustrates the corresponding analysis for MAPBr, where Figure 7e illustrates the condition used hereafter.

Table 1 shows that the lifetimes of these HBs donated by H–C(FA) are slightly smaller than for the HB donated by H–N(FA). Moreover, there is no difference between Br and I in this respect. The lifetimes of Br–H–C(MA) bonds are close to the former cases, but all of them are smaller than the Br–H–N lifetimes. This behavior has been previously noticed in ref (23) for MAPBr. Naturally, the absolute values of the lifetimes depend on the distance and angle cutoffs that define the existence of the HB. Hence, the comparison of Br–H–C and Br–H–N lifetimes is biased by the difference in the distance cutoffs. Had we set the same cutoff distance for both types of HBs, the Y–H–C HBs would have shorter lifetimes. A broader question is whether these HBs exist at all. The next section presents additional arguments.

### 3.3 Impact of HBs on the Power Spectrum

**Figure 8:**
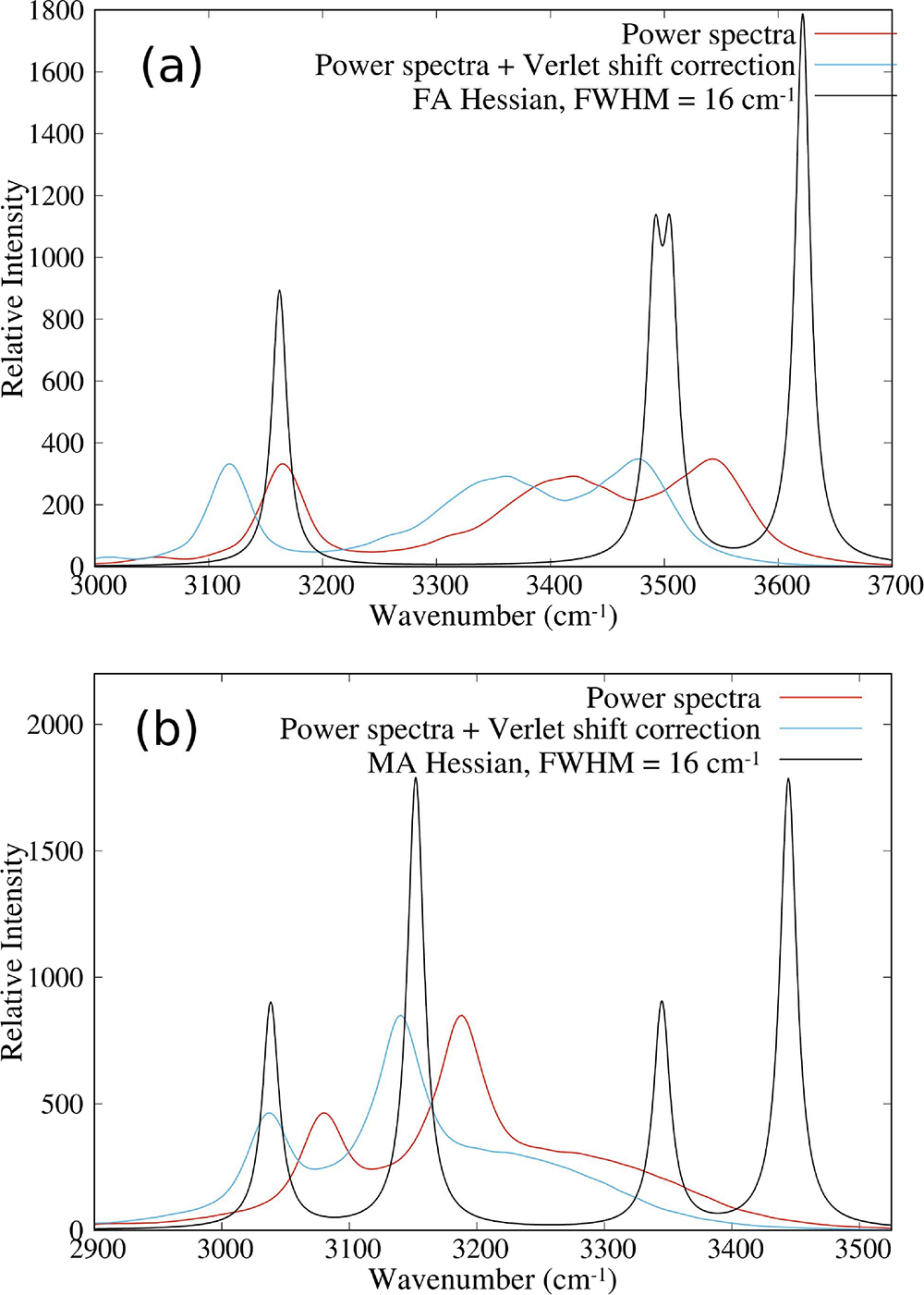
Power spectrum of FAPI (a) and MAPBr (b), compared with VDOS of isolated FA and MA obtained from the Hessian matrix.

It is well known that HBs modify the frequencies of N–H and C–H stretching vibrations^27,28^ and the shape of the power spectrum,^45^ providing experimental evidence through the IR spectra^46,47^ or Raman spectrum.^20^ Hence, we evaluate the associated power spectrum in FAPI and MAPBr. The power spectrum of MAFA has been shown to be well approximated by a weighted average of the spectra of pure compounds.^31^Figure 8 shows the power spectrum of FAPI (a) and MAPBr (b) in the range of 3000–3700 cm^–1^, compared with the vibrational density of states (VDOS) of isolated FA and MA. The frequencies of the normal modes at the minimal energy geometry were obtained by diagonalizing the Hessian of the energy as a function of the internal coordinates. The normal mode frequencies and vibration patterns are shown in Tables S3 and S4 in the Supporting Information. This method provides the power spectrum in the harmonic approximation at zero temperature. The resulting delta-like VDOS has been broadened by means of a Lorentzian function with a full width at half-maximum of 16 cm^–1^ for each vibrational mode. The peak at 3162 cm^–1^ in Figure 8a comes from the C–H stretching mode, and the peaks at higher frequencies come from N–H stretching modes. Similarly, for MA, the peak at 3038 cm^–1^ in Figure 8b comes from the symmetric stretching of C–H bonds in CH_3_, while the peak at 3152 cm^–1^ comes from two asymmetric stretching modes of C–H bonds. The two peaks at higher frequencies also come from N–H stretching modes.

The power spectra obtained from MD simulations by means of the Fourier transform of the velocity autocorrelation function are shown in red and blue lines for both FAPI and MAPBr. The blue lines include a frequency (wavenumber) correction intended to cancel a systematic error in the oscillator frequencies introduced by the finite time step in the Verlet algorithm.^41,48^ Considering the corrected curves, the vibrational frequencies at the temperature of the MD simulation (350 K) are redshifted from the zero-temperature frequencies in all cases (even in the case of the first peak in Figure 8b, there is a small redshift of 1 cm^–1^). This redshift can be understood as caused by anharmonicity and the environment. Note that peaks of the N–H stretching modes undergo larger redshifts than the C–H stretching mode peaks but also get broadened asymmetrically toward the low-frequency side. This asymmetry of the N–H stretching mode peak is a signature of the HB.^45^ The peaks of C–H stretching modes do not show asymmetric broadening, as well as none of the other peaks at frequencies under 1800 cm^–1^ (see Figure S2 in the Supporting Information). Another interesting aspect is that the power spectrum of MAPBr shown in Figure 8b displays only two peaks and one broad shoulder, while the harmonic spectrum displays four peaks. Figure S3b shows the deconvolution of this spectrum in four components, showing an excellent fit. We conclude that the two highest frequency peaks, those corresponding to the N–H stretching modes of isolated MA, get strongly broadened and redshifted, overlapping with the peaks from C–H stretching modes. A similar behavior can be appreciated, although less dramatically, in Figure 8a for FAPI, whose power spectrum can be fitted with five components (Figure S3a). Furthermore, the lowest frequency peaks in Figure 8, corresponding to C–H stretching modes, are well fitted with Lorentzian functions, while the fitting functions centered at higher frequencies are Gaussians. All this supports the interpretation that C–H stretching modes of FA and MA are rather well preserved in the perovskites, but the N–H stretching modes are strongly modified. We conclude that this is an observable effect of HBs donated by the N–H groups in both FA and MA. Our analysis also suggests that the C–H–Y HBs, if they exist at all, have little effect on vibrational properties.

### 3.4 Electronic Structure

HBs and other non-covalent interactions (NCIs) can be proved in electronic structure calculations by means of the reduced density gradient (RDG).^29^ Varadwaj et al.^21^ have shown that the RDG-NCI analysis supports the existence of HBs in MAPI. Here, we show that their results also apply to FAPI and MAPBr. The RDG is defined as *s* = 1/(2(3π^2^)^1/3^)| ∇ ρ|/ρ^4/3^, where ρ is the electron density. As commented above, one of the IUPAC criteria for HBs is the existence of a (3, −1) critical point along the Y–H path. In a (3, −1) point, the RDG is null, and the Hessian matrix of the density has two negative eigenvalues and one positive eigenvalue, ordered as λ_1_ ≤ λ_2_ ≤ λ_3_. Hence, the negative sign of λ_2_ (as for λ_1_ ≤ λ_2_) where *s* = 0 indicates a (3, −1) critical point. Low values of the density indicate NCIs, in contrast with covalent bonds associated with larger values of the electron density. Hence, there is a certain range of values of the pair (*s*, sign (λ_2_)ρ) that is typical in the vicinity of the (3, −1) critical point of HBs. This range is approximately *s* ∈ (0.2,0.5) and sign(λ_2_)ρ ∈ ( – 0.05, – 0.01).^21,29^

**Figure 9:**
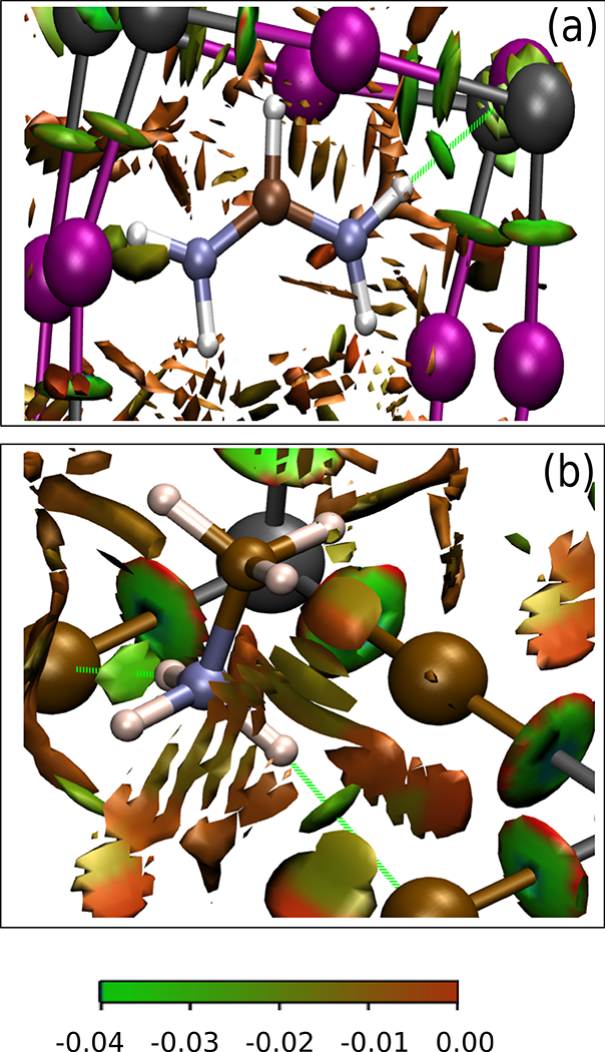
RDG plots in FAPI (a) and MAPBr (b), revealing NCIs. HBs are signaled by green clouds near the centers of the H–I and H–Br paths, appearing only when the geometrical conditions of HBs are fulfilled.

Figure 9 shows the RDG isosurfaces in unit cells of FAPI (a) and MAPBr (b). The isosurfaces *s*(*x*, *y*, *z*) = 0.5 are shown, using a color scale graded from the function sign(λ_2_)ρ. The calculations were made using Gaussian09^49^ for non-periodic clusters FA_19_Pb_8_I_36_ and MA_19_Pb_8_Br_36_, both with charge −1 to have a closed shell electronic structure. The PBE functional was used with the LANL2DZ basis set. The cluster of FAPI was cut from a configuration of the MD ensemble that has one HB minimal I–H(N) distance (2.27 Å), while the cluster of MAPBr was cut from a relaxed periodic structure with a single unit cell, having two HBs with the Br–H(N) distance equal to 2.43 Å and 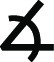
(Br – H – N) = 168°. The regions shown in Figure 9 belong to the central unit cell of each cluster. For both FAPI and MAPBr, we can appreciate a small green cloud (sign(λ_2_)ρ ≈ – 0.04) in the middle of each HB (green broken lines), but no similar cloud can be seen near the other hydrogens that do not fulfill the HB geometrical conditions. It is particularly interesting that MAPBr does not show any (3, −1) critical point connecting the third H atom of the ammonium fragment with the nearest Br atom at 2.61 Å. This H atom narrowly misses the HB geometrical criterion because 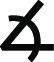
(Br – H – N) = 132.5°.

## 4 Discussion and Conclusions

Let us now discuss some recent publications about HBs in HOIHPs in the context of our results. Saleh et al.^20^ have conducted a classical MD study with a huge number of atoms. They concluded that the HBs control the energetics of MA orientations. As they did not consider the directional criterion^27^ in their geometrical definition of the HB, they obtained HB populations that are much greater than our estimations. On the opposite side, Ibaceta-Jaña et al.^26^ have recently disputed the presence of HBs in halide perovskites at room temperature. They proved HBs using Raman spectroscopy, comparing signatures of HBs in water, MAI, and FAI with the spectra on MAPbY_3_ and FAPbY_3_. The absence of spectroscopic evidence does not prove the inexistence of HBs but rather proves its irrelevance. However, the failure to observe HB signatures in their Raman spectra can be attributed to the fact that they centered the analysis in the range of 500–1800 cm^–1^, where our simulations also do not show appreciable effects. According to our simulations (Figure 8), HBs influence vibrational properties only for the stretching modes N–H over 3000 cm^–1^, agreeing with ref (26) in the absence of Raman signatures for lower-frequency modes. Moreover, the Raman spectra for MAPBr in the range of 3000–3500 cm^–1^ reported in the Supporting Information of ref (26) seem to agree with our simulations regarding the disappearance of the peaks associated to N–H stretching modes. In any event, the HBs in HOIHPs are very weak.

How could we contrast the short HB lifetimes with experimental evidence? Mozur and Neilson^50^ have compiled a long list of activation barriers and residence times associated to the movement of organic cations in different HOIHPs. Residence times associated to wobble-in-a-cone motion of MA could be related with the HB lifetimes, although an exact correspondence cannot be established. These residence times at room temperature (∼0.3 ps) are of the same order of magnitude as our HB lifetimes. These characteristic times show up in rotational time correlation functions obtained from MD simulations.^51^ The difference with the lifetime of the HBs can be that the latter can break and form again before a wobbling rotation takes place. To the best of our knowledge, no direct measurements of HB lifetimes have been made.

In summary, in this study, we have studied the HBs in three halide perovskites by analyzing MD simulations. These HBs have been defined from geometrical criteria as a function of the distance and angle between the atoms. The presented geometrical criteria are based on the angle–distance CDF and the IUPAC recommendations. We have characterized the statistical properties of the HBs by means of PDF, CDF, and time correlation functions. From the latter, we obtained HB lifetimes, which are smaller for C–H–Y bonds than for N–H–Y bonds (Y = Br and I). The HB lifetimes are similar in the solid solution (FAPbI_3_)_7/8_(MAPbBr_3_)_1/8_ and in the end compounds FAPbI_3_ and MAPbBr_3_.

On the other hand, analyzing the power spectrum, it has been possible to verify that the power spectra peaks related to N–H stretching modes, especially in MA, are modified as expected from the influence of HBs. However, this is not the case for the C–H stretching modes (Figure 8). This analysis suggests that C–H–Y HBs are irrelevant for the vibrational spectra. The HBs in these compounds are weak and possibly undetectable at the high temperature that was set in the MD simulations. HBs should manifest at lower temperatures, particularly near the phase transitions. Our simulations were performed at 350 K, but this work establishes a theoretical and computational framework for the investigation of HBs that can be applied for the analysis of MD simulations at other temperatures.

## Supporting Information Available

Document including description of animations, additional CDFs, definition of the HB autocorrelation function, assignment of vibration modes, extended range and deconvolution of power spectra (PDF)
Animation of HB dynamics for I–H–C(FA) and I–H–N(FA) in FAPI (MP4)
Animation of HB dynamics for Br–H–C(MA) and Br–H–N(MA) in MAPBr (MP4)
Animation of HB dynamics for I–H–C(FA–MA–MA) and I–H–N(FA–MA–MA) in MAFA (MP4)
Animation of HB dynamics for I–H–C(FA–MA–FA) and I–H–N(FA–MA–FA) in MAFA (MP4)
Animation of HB dynamics for Br–H–C(FA–MA–MA) and Br–H–N(FA–MA–MA) in MAFA (MP4)
Animation of HB dynamics for Br–H–C(FA–MA–FA) and Br–H–N(FA–MA–FA) in MAFA (MP4)
Animation of HB dynamics in FAPI with HBs defined without angular condition (MP4)
Animation of HB dynamics in FAPI with HBs wrong angular condition (MP4)
Excel spreadsheet with raw data obtained from TRAVIS: CDFs, autocorrelation functions, and lifetimes (XLSX)

## References

[ref1] Kojima A.; Teshima K.; Shirai Y.; Miyasaka T. Organometal Halide Perovskites as Visible-Light Sensitizers for Photovoltaic Cells. J. Am. Chem. Soc. 2009, 131, 6050–6051. 10.1021/ja809598r. 19366264

[ref2] NREL chart on record cell efficiencies. https://www.nrel.gov/pv/cell-efficiency.html (accessed June 8, 2023).

[ref3] Wang Y.; Lou H.; Yue C.-Y.; Lei X.-W. Applications of halide perovskites in X-ray detection and imaging. CrystEngComm 2022, 24, 2201–2212. 10.1039/D1CE01575C.

[ref4] Kim H.; Zhao L.; Price J. S.; Grede A. J.; Roh K.; Brigeman A. N.; Lopez M.; Rand B. P.; Giebink N. C. Hybrid perovskite light emitting diodes under intense electrical excitation. Nat. Commun. 2018, 9, 4893 10.1038/s41467-018-07383-8. 30459326 PMC6244086

[ref5] Huang H.; Pradhan B.; Hofkens J.; Roeffaers M. B. J.; Steele J. A. Solar-driven metal halide perovskite photocatalysis: design, stability, and performance. ACS Energy Lett. 2020, 5, 1107–1123. 10.1021/acsenergylett.0c00058.

[ref6] Ono L. K.; Juarez-Perez E. J.; Qi Y. Progress on Perovskite Materials and Solar Cells with Mixed Cations and Halide Anions. ACS Appl. Mater. Interfaces 2017, 9, 30197–30246. 10.1021/acsami.7b06001. 28682587

[ref7] Walsh A.; elds22; Brivio F.; Frost J. M. WMD-group/hybrid-perovskites: Collection 1; Zenodo, April 16, 2019, 10.5281/zenodo.2641358.

[ref8] Momma K.; Izumi F. VESTA 3 for three-dimensional visualization of crystal, volumetric and morphology data. J. Appl. Crystallogr. 2011, 44, 1272–1276. 10.1107/S0021889811038970.

[ref9] Lee J.-W.; Tan S.; Seok S. I.; Yang Y.; Park N.-G. Rethinking the A cation in halide perovskites. Science 2022, 375, eabj1186 10.1126/science.abj1186. 35201885

[ref10] Stoumpos C. C.; Malliakas C. D.; Kanatzidis M. G. Semiconducting Tin and Lead Iodide Perovskites with Organic Cations: Phase Transitions, High Mobilities, and Near-Infrared Photoluminescent Properties. Inorg. Chem. 2013, 52, 9019–9038. 10.1021/ic401215x. 23834108

[ref11] Cheng Z.; Lin J. Layered organic–inorganic hybrid perovskites: structure, optical properties, film preparation, patterning and templating engineering. CrystEngComm 2010, 12, 2646–2662. 10.1039/C001929A.

[ref12] Francisco-López A.; Charles B.; Alonso M. I.; Garriga M.; Campoy-Quiles M.; Weller M. T.; Goñi A. R. Phase Diagram of Methylammonium/Formamidinium Lead Iodide Perovskite Solid Solutions from Temperature-Dependent Photoluminescence and Raman Spectroscopies. J. Phys. Chem. C 2020, 124, 3448–3458. 10.1021/acs.jpcc.9b10185.

[ref13] Onoda-Yamamuro N.; Matsuo T.; Suga H. Calorimetric and IR spectroscopic studies of phase transitions in methylammonium trihalogenoplumbates (II)†. J. Phys. Chem. Solids 1990, 51, 1383–1395. 10.1016/0022-3697(90)90021-7.

[ref14] Senno M.; Tinte S. Mixed formamidinium–methylammonium lead iodide perovskite from first-principles: hydrogen-bonding impact on the electronic properties. Phys. Chem. Chem. Phys. 2021, 23, 7376–7385. 10.1039/D0CP06713J. 33876097

[ref15] Carignano M. A.; Kachmar A.; Hutter J. Thermal Effects on CH_3_NH_3_PbI_3_ Perovskite from Ab Initio Molecular Dynamics Simulations. J. Phys. Chem. C 2015, 119, 8991–8997. 10.1021/jp510568n.

[ref16] Huang B.; Liu Z.; Wu C.; Zhang Y.; Zhao J.; Wang X.; Li J. Polar or nonpolar? That is not the question for perovskite solar cells. Natl. Sci. Rev. 2021, 8 10.1093/nsr/nwab094. PMC8363338 34691717

[ref17] Montero-Alejo A. L.; Menéndez-Proupin E.; Hidalgo-Rojas D.; Palacios P.; Wahnón P.; Conesa J. C. Modeling of Thermal Effect on the Electronic Properties of Photovoltaic Perovskite CH_3_NH_3_PbI_3_: The Case of Tetragonal Phase. J. Phys. Chem. C 2016, 120, 7976–7986. 10.1021/acs.jpcc.6b01013.

[ref18] Lodeiro L.; Barría-Cáceres F.; Jiménez K.; Contreras R.; Montero-Alejo A. L.; Menéndez-Proupin E. Methodological Issues in First-Principle Calculations of CH_3_NH_3_PbI_3_ Perovskite Surfaces: Quantum Confinement and Thermal Motion. ACS Omega 2020, 5, 29477–29491. 10.1021/acsomega.0c04420. 33225179 PMC7676347

[ref19] Mosconi E.; De Angelis F. Mobile Ions in Organohalide Perovskites: Interplay of Electronic Structure and Dynamics. ACS Energy Lett. 2016, 1, 182–188. 10.1021/acsenergylett.6b00108.

[ref20] Saleh G.; Biffi G.; Di Stasio F.; Martín-García B.; Abdelhady A. L.; Manna L.; Krahne R.; Artyukhin S. Methylammonium Governs Structural and Optical Properties of Hybrid Lead Halide Perovskites through Dynamic Hydrogen Bonding. Chem. Mater. 2021, 33, 8524–8533. 10.1021/acs.chemmater.1c03035.

[ref21] Varadwaj P. R.; Varadwaj A.; Marques H. M.; Yamashita K. Significance of hydrogen bonding and other noncovalent interactions in determining octahedral tilting in the CH_3_NH_3_PbI_3_ hybrid organic-inorganic halide perovskite solar cell semiconductor. Sci. Rep. 2019, 9, 50 10.1038/s41598-018-36218-1. 30631082 PMC6328624

[ref22] Mitzi D. B. Templating and structural engineering in organic–inorganic perovskites. J. Chem. Soc., Dalton Trans. 2001, 1–12. 10.1039/B007070J.

[ref23] Maity S.; Verma S.; Ramaniah L. M.; Srinivasan V. Deciphering the Nature of Temperature-Induced Phases of MAPbBr_3_ by Ab Initio Molecular Dynamics. Chem. Mater. 2022, 34, 10459–10469. 10.1021/acs.chemmater.2c02453.

[ref24] Millikan R. A. On the Elementary Electrical Charge and the Avogadro Constant. Phys. Rev. 1913, 2, 109–143. 10.1103/PhysRev.2.109.

[ref25] Svane K. L.; Forse A. C.; Grey C. P.; Kieslich G.; Cheetham A. K.; Walsh A.; Butler K. T. How Strong Is the Hydrogen Bond in Hybrid Perovskites?. J. Phys. Chem. Lett. 2017, 8, 6154–6159. 10.1021/acs.jpclett.7b03106. 29216715 PMC5765532

[ref26] Ibaceta-Jaña J.; Chugh M.; Novikov A. S.; Mirhosseini H.; Kühne T. D.; Szyszka B.; Wagner M. R.; Muydinov R. Do Lead Halide Hybrid Perovskites Have Hydrogen Bonds?. J. Phys. Chem. C 2022, 126, 16215–16226. 10.1021/acs.jpcc.2c02984.

[ref27] Arunan E.; Desiraju G. R.; Klein R. A.; Sadlej J.; Scheiner S.; Alkorta I.; Clary D. C.; Crabtree R. H.; Dannenberg J. J.; Hobza P.; et al. Definition of the hydrogen bond (IUPAC Recommendations 2011). Pure Appl. Chem. 2011, 83, 1637–1641. 10.1351/PAC-REC-10-01-02.

[ref28] Arunan E.; Desiraju G. R.; Klein R. A.; Sadlej J.; Scheiner S.; Alkorta I.; Clary D. C.; Crabtree R. H.; Dannenberg J. J.; Hobza P.; et al. Defining the hydrogen bond: An account (IUPAC Technical Report). Pure Appl. Chem. 2011, 83, 1619–1636. 10.1351/PAC-REP-10-01-01.

[ref29] Johnson E. R.; Keinan S.; Mori-Sánchez P.; Contreras-García J.; Cohen A. J.; Yang W. Revealing Noncovalent Interactions. J. Am. Chem. Soc. 2010, 132, 6498–6506. 10.1021/ja100936w. 20394428 PMC2864795

[ref30] Lee J. H.; Lee J.-H.; Kong E.-H.; Jang H. M. The nature of hydrogen-bonding interaction in the prototypic hybrid halide perovskite, tetragonal CH_3_NH_3_PbI_3_. Sci. Rep. 2016, 6, 21687 10.1038/srep21687. 26892429 PMC4759593

[ref31] Menéndez-Proupin E.; Grover S.; Montero-Alejo A. L.; Midgley S. D.; Butler K. T.; Grau-Crespo R. Mixed-anion mixed-cation perovskite (FAPbI_3_)_0.875_(MAPbBr_3_)_0.125_: an ab initio molecular dynamics study. J. Mater. Chem. A 2022, 10, 9592–9603. 10.1039/D1TA10860C.

[ref32] Menéndez-Proupin E.; Grover S.; Montero-Alejo A. L.; Midgley S. D.; Butler K. T.; Grau-Crespo R. Data supporting Mixed-anion mixed-cation perovskite (FAPbI3)0.875(MAPbBr3)0.125: an ab initio molecular dynamics study [Data set]; Zenodo, Nov 19, 2021, 10.5281/zenodo.8006481.

[ref33] Kühne T. D.; Iannuzzi M.; Del Ben M.; Rybkin V. V.; Seewald P.; Stein F.; Laino T.; Khaliullin R. Z.; Schütt O.; Schiffmann F.; et al. CP2K: An electronic structure and molecular dynamics software package - Quickstep: Efficient and accurate electronic structure calculations. J. Chem. Phys. 2020, 152, 194103 10.1063/5.0007045. 33687235

[ref34] Perdew J. P.; Burke K.; Ernzerhof M. Generalized Gradient Approximation Made Simple. Phys. Rev. Lett. 1996, 77, 3865–3868. 10.1103/PhysRevLett.77.3865. 10062328

[ref35] Grimme S.; Antony J.; Ehrlich S.; Krieg H. A consistent and accurate ab initio parametrization of density functional dispersion correction (DFT-D) for the 94 elements H-Pu. J. Chem. Phys. 2010, 132, 154104 10.1063/1.3382344. 20423165

[ref36] VandeVondele J.; Krack M.; Mohamed F.; Parrinello M.; Chassaing T.; Hutter J. Quickstep: Fast and accurate density functional calculations using a mixed Gaussian and plane waves approach. Comput. Phys. Commun. 2005, 167, 103–128. 10.1016/j.cpc.2004.12.014.

[ref37] VandeVondele J.; Hutter J. Gaussian basis sets for accurate calculations on molecular systems in gas and condensed phases. J. Chem. Phys. 2007, 127, 114105 10.1063/1.2770708. 17887826

[ref38] Goedecker S.; Teter M.; Hutter J. Separable dual-space Gaussian pseudopotentials. Phys. Rev. B: Condens. Matter 1996, 54, 1703–1710. 10.1103/PhysRevB.54.1703. 9986014

[ref39] Hartwigsen C.; Goedecker S.; Hutter J. Relativistic separable dual-space Gaussian pseudopotentials from H to Rn. Phys. Rev. B: Condens. Matter 1998, 58, 3641–3662. 10.1103/PhysRevB.58.3641. 9986014

[ref40] Krack M. Pseudopotentials for H to Kr optimized for gradient-corrected exchange-correlation functionals. Theor. Chem. Acc. 2005, 114, 145–152. 10.1007/s00214-005-0655-y.

[ref41] Brehm M.; Thomas M.; Gehrke S.; Kirchner B. TRAVIS—A free analyzer for trajectories from molecular simulation. J. Chem. Phys. 2020, 152, 164105 10.1063/5.0005078. 32357781

[ref42] Humphrey W.; Dalke A.; Schulten K. V. M. D. Visual molecular dynamics. J. Mol. Graph. 1996, 14, 33–38. 10.1016/0263-7855(96)00018-5. 8744570

[ref43] Alder R. W.; Blake M. E.; Oliva J. M. Diaminocarbenes; Calculation of Barriers to Rotation about C_carbene_–N Bonds, Barriers to Dimerization, Proton Affinities, and ^13^C NMR Shifts. J. Phys. Chem. A 1999, 103, 11200–11211. 10.1021/jp9934228.

[ref44] Teunissen J. L.; Da Pieve F. Molecular Bond Engineering and Feature Learning for the Design of Hybrid Organic–Inorganic Perovskite Solar Cells with Strong Noncovalent Halogen–Cation Interactions. J. Phys. Chem. C 2021, 125, 25316–25326. 10.1021/acs.jpcc.1c07295.

[ref45] Brehm M.; Sebastiani D. Simulating structure and dynamics in small droplets of 1-ethyl-3-methylimidazolium acetate. J. Chem. Phys. 2018, 148, 193802 10.1063/1.5010342. 30307180

[ref46] Wallace W. E. Infrared Spectra: Phenol Condensed Phase Spectrum: NIST Mass Spectrometry Data Center, NIST Chemistry WebBook, NIST Standard Reference Database *Number 69, Eds.* Linstrom P. J.; Mallard W. G.. National Institute of Standards and Technology: Gaithersburg MD, 20899, 2018. https://webbook.nist.gov/cgi/cbook.cgi?ID=C108952&Units=SI&Type=IR-SPEC&Index=1 (accessed 2023 February 1).

[ref47] Wallace W. E. Infrared Spectra: Phenol Gas phase spectrum, NIST Chemistry WebBook, NIST Standard Reference Database Number 69*, Eds.* Linstrom P. J.; Mallard W. G.. National Institute of Standards and Technology: Gaithersburg MD, 20899, 2018. https://webbook.nist.gov/cgi/cbook.cgi?ID=C108952&Type=IR-SPEC&Index=0 (accessed 2023 February 1).

[ref48] Thomas M. Theoretical Modeling of Vibrational Spectra in the Liquid Phase; Springer Cham, 2016. 10.1007/978-3-319-49628-3.

[ref49] Frisch M. J.; Trucks G. W.; Schlegel H. B.; Scuseria G. E.; Robb M. A.; Cheeseman J. R.; Scalmani G.; Barone V.; Mennucci B.; Petersson G. A.; Gaussian 09 Rev. B.01; Gaussian Inc.: Wallingford, CT, 2009.

[ref50] Mozur E. M.; Neilson J. R. Cation Dynamics in Hybrid Halide Perovskites. Annu. Rev. Mater. Res. 2021, 51, 269–291. 10.1146/annurev-matsci-080819-012808.

[ref51] Selig O.; Sadhanala A.; Müller C.; Lovrincic R.; Chen Z.; Rezus Y. L. A.; Frost J. M.; Jansen T. L. C.; Bakulin A. A. Organic Cation Rotation and Immobilization in Pure and Mixed Methylammonium Lead-Halide Perovskites. J. Am. Chem. Soc. 2017, 139, 4068–4074. 10.1021/jacs.6b12239. 28240902

