# Supplementary material for: Hydrogen Bonds in Lead Halide Perovskites: Insights from Ab Initio Molecular Dynamics
Source: J Phys Chem C Nanomater Interfaces. 2023 Aug 8;127(32):15901–10. doi: 10.1021/acs.jpcc.3c02376 (PMC10440809; doi:10.1021/acs.jpcc.3c02376)
Supplement: Supplementary file 10 — jp3c02376_si_010.pdf [file jp3c02376_si_010.pdf]

SUPPORTING INFORMATION

Hydrogen Bonds in Lead Halide Perovskites: Insights from *Ab Initio* Molecular Dynamics

Alejandro Garrote-Márquez,[a] Lucas Lodeiro,[b] Rahul Suresh,[a,c] Norge Cruz Hernández,[a] Ricardo Grau-Crespo,[d] and Eduardo Menéndez-Proupin[a]

[a] *Departamento de Física Aplicada I, Escuela Politécnica Superior, Universidad de Sevilla, Seville E-41011, Spain.*

[b] *Departamento de Química, Facultad de Ciencias, Universidad de Chile, Las Palmeras 3425, Nuñoa 7800003, Santiago, Chile*

[c] *International Research Center of Spectroscopy and Quantum Chemistry - IRC SQC, Siberian Federal University, 79 Svobodny pr., 660041 Krasnoyarsk, Russia*

[d] *Department of Chemistry, University of Reading, Whiteknights, Reading RG6 6DX, UK.*

Explanation of videos

Videos V1-V6 illustrate the dynamic formation and breaking of hydrogen bonds (HBs) donated by formamidinium (FA) and methylammonium (MA) in the compounds in $MAPbBr_3$ (MAPBr), $FAPbI_3$ (FAPI) and the solid solution $(FAPbI_3)_{7/8}(MAPbBr_3)_{1/8}$ (MAFA) respectively. In these animations, the HBs are defined by the simultaneous fulfillment of the conditions $d(\mathrm{Y-N}) < 4$ Å or $d(\mathrm{Y-C}) < 5$ Å, and $\measuredangle(\mathrm{Y-H-X}) > 135°$, where X=C, N, and Y=Br, I. This means that the VMD program draws a dotted stick along the Y—H path when both conditions are fulfilled. Videos V7 and V8 illustrate the effect of eliminating or relaxing the angular condition. Table S1 explain explains the labelling of each video.

Table S1. Videos showing the formation and breaking of HB X-H—Y.

Label and filename	Content
V1 V1_I--H-C_FA_and_I--H-N_FA.mp4	FA motion in FAPI showing N-H—I and C-H—I HBs.
V2 V2_Br--H-C_MA_and_Br--H-N_MA.mp4	MA motion in MAPBr showing N-H—Br and C-H—Br HBs.
V3 V3_I--H-C_FA-MA_MA_and_I--H-N_FA-MA_MA.mp4	MA motion in MAFA showing N-H—I and C-H—I HBs.
V4 V4_I--H-C_FA-MA_FA_and_I--H-N_FA-MA_FA.mp4	FA motion in MAFA showing N-H—I and C-H—I HBs.
V5 V5_Br--H-C_FA-MA_MA_and_Br--H-N_FA-MA_MA.mp4	MA motion in MAFA showing N-H—Br and C-H—Br HBs.
V6 V6_Br--H-C_FA-MA_FA_and_Br--H-N_FA-MA_FA.mp4	FA motion in MAFA showing N-H—Br and C-H—Br HBs.
V7 V7_I--N-vs-HB.mp4	Illustration of multiple (fake) HBs in FAPI when defined just by condition $d(N-I) < 4$ Å
V8 V8_I--H-C_FA_and_I--H-N_FA_wrong-condition.mp4	Illustration of multiple (fake) C-H—I HBs in FAPI when defined by $d(N-I) < 4$ Å, $90° < \measuredangle(I-H-C) < 180°$. To be compared with V1.

Alternative definition of HB from nitrogen-halide distance

Figure S1 complements **Figure 5** of the main text. The snapshots in second and fourth columns in **Figure S1** show the Y—N less than 4 Å (sticks in gray color) together with HBs (green color). This illustrates that the first PDF Y—N peak seen in the PDF of **Figure 3** is not directly related to HBs. However, the region in the combined distribution function (CDF) corresponding to $\measuredangle(Y-H-N) > 135°$ shows a depletion for the distance between 4 and 6 Å for all compounds. This the signature of HB in the CDF. This fact rules out the other zones of maxima that are observed in the CDF whose angles are less than 135º.

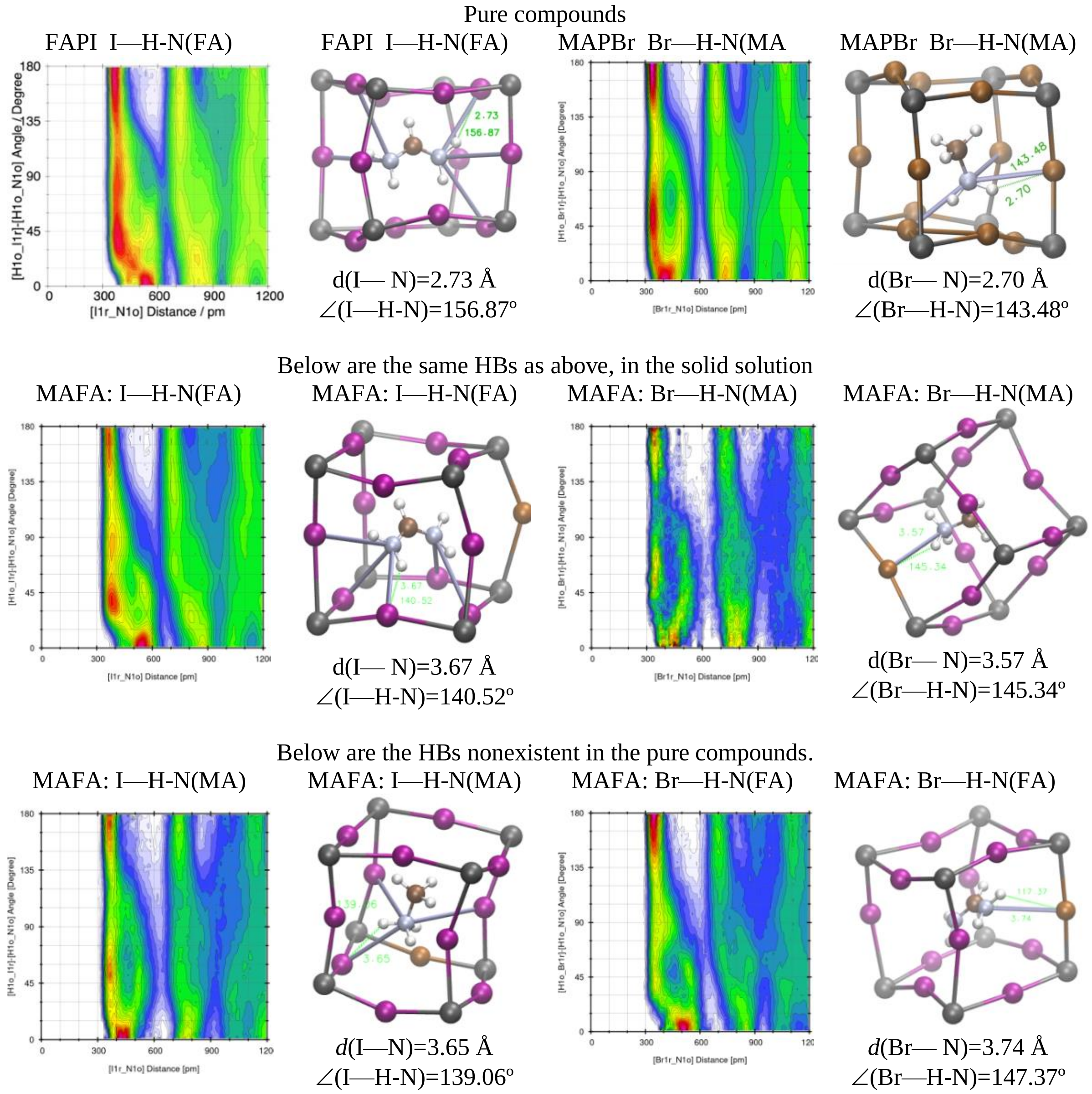

Figure S1. Combined distribution function of the Y—N distance and Y—H—N angle in FAPI, MAPBr, and the solid solution MAFA. Snapshots of the MD ensembles illustrating one HB (green dotted line) and the fake HB defined only from Y—N distances (solid lines).

Lifetimes of HBs

The duration of HBs is characterized by the continuous time correlation function defined as

$$C_C^{HB}(t) = \frac{\langle h(0)H(t)\rangle - \langle h\rangle\langle H\rangle}{\langle h\rangle - \langle h\rangle\langle H\rangle},$$

Where h is unity when a particular HB is formed (according to the geometrical conditions specified) and zero otherwise, and $H(t)$ is unity of the tagged HB has been continuously formed during a time t. The average $\langle ... \rangle$ is taken over all the HBs and over all time origins in the simulation. The lifetimes derived from the autocorrelation functions are presented in Table S1.

The datasheet AGGR-CDF.xlsx contains the data obtained from the TRAVIS code for each type of HB. In the process to obtain the CDFs and the autocorrelation functions, the TRAVIS program labels all the halides as equivalent species, i.e., I1, Br1, and all FA or MA as the same species as well. However, for each cation its atoms are labeled according to its position at the cation backbone. Thus, each FA contains N1, N2, and C1, while each MA has N1 and C1. The hydrogens of the cations are: H1-H5 from FA (H1 and H2 bound to N1, H3 and H4 bound to N2, H5 bound to C1), and H1-H6 from MA (H1-H3 bound to N1, H4-H6 bound to C1). Each cation in the supercell has one of these species and TRAVIS calculates the functions averaged for each species. We have obtained the CDF and the autocorrelation functions defining each triple X-H—Y. For example, for the HBs N-H—I in FAPI we have defined the four triples N1-H1—I, N1-H2—I (H1 and H2 connected with N1), N2-H3—I, N2-H4—I (H3 and H4 connected with N2). The plots of the CDFs and autocorrelation functions, and the lifetimes for each triplet are shown in the data sheet AGGR-CDF.xlsx. **Table S1** summarizes the HB lifetimes for each of these triples. Next, we have averaged the correlation functions, and lifetimes for the triples that are equivalent, and those are the results reported in the main text.

Table S2. Lifetimes (in ps) of the different types of HBs derived from the HB continuous time correlation functions. One lifetime for every hydrogen covalently bond to a nitrogen atom is given. The H bound to either of the two N atoms in FA are signaled with red and green color.

	FAPI	MAPBr	MAFA
I—H-N(MA)			0.20,0.15, 0.18
Br—H-N(MA)		0.22,0.23,0.23	0.24,0.23,0.34
I—H-C(MA)			0.14,0.14,0.14
Br—H-C(MA)		0.16,0.16,0.16	0.17,0.14,0.14
I—H-C(FA)	0.15		0.16
Br—H-C(FA)			0.18
I—H-N(FA)	0.15,0.15,0.15,0.15		0.16,0.14,0.15,0.16
Br—H-N(FA)			0.21,0.23,0.22,0.25

Power spectra

Figure S2 shows the power spectrum of FAPI (a, b) and MAPBr (c, d) for the range of low and high frequencies spanning the vibration modes of the organic cations. The highest frequency range, over 3000 cm^{-1}, corresponds to C-H and N-H stretching modes. The vibrational density of states (VDOS) of isolated FA and MA are also shown, as computed from diagonalization of the Hessian of the energy. **Tables S3** and **S4** show the assignments of these modes.

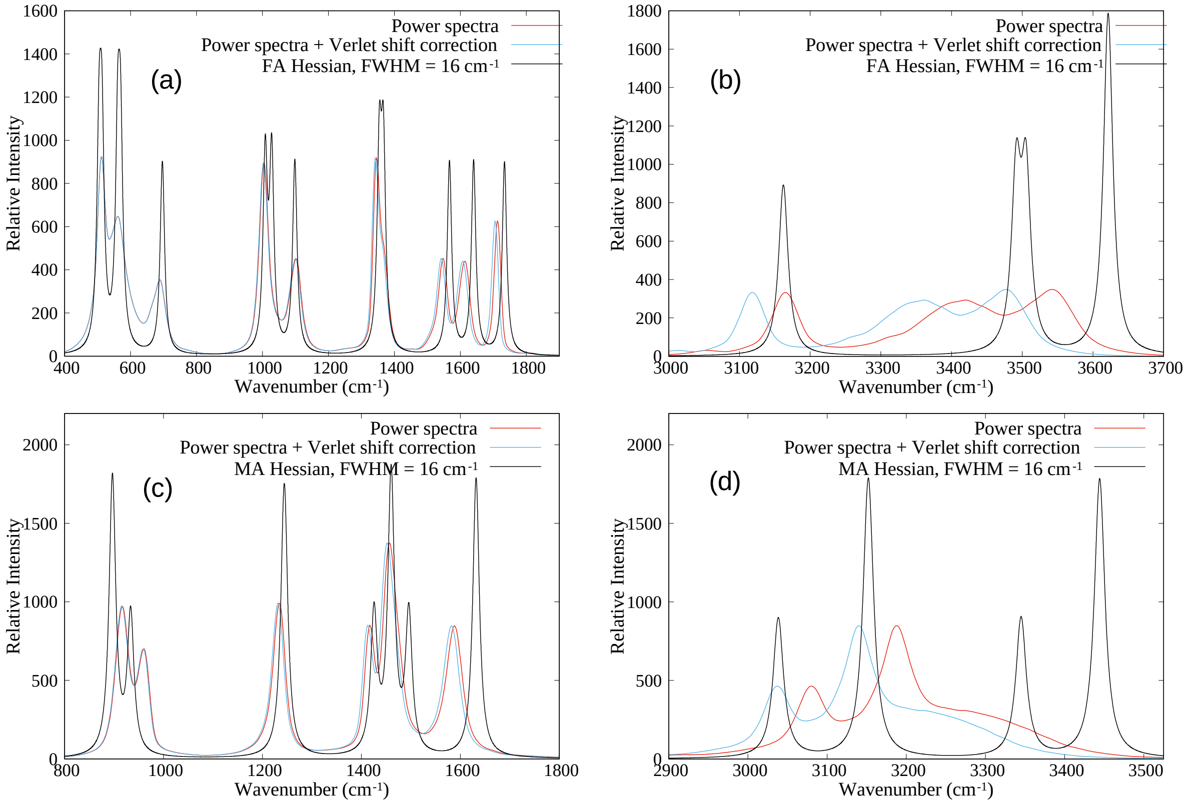

Figure S2. Power spectrum of FAPI (a, b) and MAPBr (c, d) and vibrational DOS of isolated FA and MA.

Table S3. DFT calculated X-H stretching modes of the isolated FA^+ cation. C_{2v} symmetry group. Only large H displacements are indicated with arrows.

N°	Mode Description	Irred. Rep.	Freq. (cm^{-1})	Image
14	CH stretching	A_1	3162.1	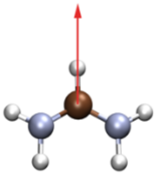
15	NH_2 sym. stretching	B_1	3491.6	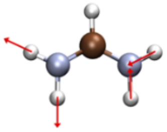
16	NH_2 sym. stretching	A_1	3505.3	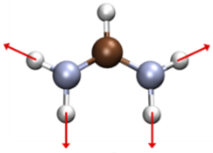
17	NH_2 asym. stretching	B_1	3621.3	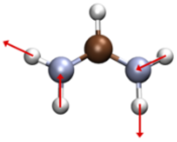
18	NH_2 asym. stretching	A_1	3621.9	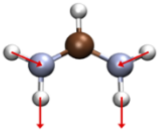

Table S4. DFT calculated X-H stretching modes of the isolated MA^+ cation. C_{3v} symmetry group. Only large H displacements are indicated with arrows.

N°	Mode Description	Irred. Rep.	Freq. (cm^{-1})	Image
13	CH_3 sym. stretching	A_1	3038.4	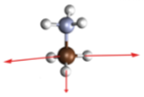
14	CH_3 asym. stretching	E	3152.0	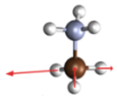
15	CH_3 asym. stretching	E	3152.1	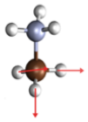
16	NH_3 sym. stretching	A_1	3345.0	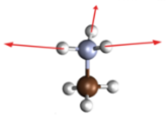
17	NH_3 asym. stretching	E	3443.8	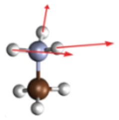
18	NH_3 asym. stretching	E	3444.6	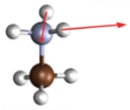

It is interesting that the power spectrum of MAPBr shown in **Figure S2d** displays only two peaks and one broad shoulder, while the harmonic approximation displays four peaks. **Figure S3b** shows the deconvolution of this spectrum into four components, with an excellent fit. We believe that the two highest frequency peaks, corresponding to the N-H stretching modes of isolated MA, get strongly broadened and redshifted, overlapping with the peaks from C-H stretching modes. The same behavior can be appreciated in FAPI, whose power spectrum can be fitted with five components (**Figure S3a**). In these fitting curves the lowest frequency peaks, corresponding to C-H stretching modes, are fitted with Lorentzian functions, while the functions centered at higher frequencies are Gaussians. This analysis supports the interpretation that C-H stretching modes of FA and MA are rather well maintained in the perovskites, but the N-H stretching modes are modified and mixed due to interaction with the surrounding atoms.

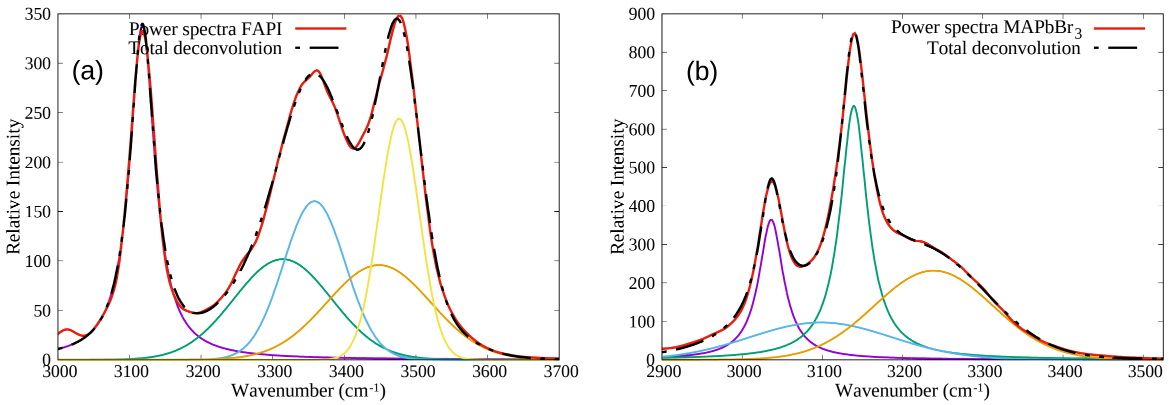

Figure S3. Deconvolution of the power spectrum of a) FAPI and b) MAPBr.

The number of fitting functions in the deconvolution was guided by the number of vibrational frequencies. The best fits have been obtained combining Lorentzian and Gaussian functions for C-H stretching modes and N-H stretching modes, respectively. We also tried fitting with all Lorentzians and all Gaussians. For instance, in the case of FAPI we used five functions: all Gaussians (G5), all Lorentzians (L5) and one Lorentzian with 4 Gaussians (L1G4). The RMS of the residuals are: 12.1454 (G5), 10.3376 (L5) and 5.29813 (L1G4). For MAPBr we used four functions, considering just one function for each of pair of E-symmetry modes. The obtained RMS of residuals are: 25.0294 (G4), 11.7787 (L4) and 5.29835 (L2G2). In both cases the mixed Lorentzian-Gaussian deconvolution provides the lowest RMS of the residuals, in agreement with visual evaluation. Gaussian broadening indicates random fluctuations in the vibrational frequencies.
